# Anti-Cancer Effects of *Lactobacillus plantarum* L-14 Cell-Free Extract on Human Malignant Melanoma A375 Cells

**DOI:** 10.3390/molecules25173895

**Published:** 2020-08-26

**Authors:** Jaehyun Park, Mijin Kwon, Jaehoon Lee, Sangkyu Park, Jeongmin Seo, Sangho Roh

**Affiliations:** 1 Cellular Reprogramming and Embryo Biotechnology Laboratory, Dental Research Institute, BK21, Seoul National University School of Dentistry, Seoul 08826, Korea; 680087@hanmail.net (J.P.); rnjsalwls23@hanmail.net (M.K.); 2 Biomedical Research Institute, NeoRegen Biotech Co., Ltd., Gyeonggi-do 16614, Korea; jaylee6322@gmail.com (J.L.); good0039@hanmail.net (S.P.)

**Keywords:** melanoma, *Lactobacillus plantarum*, cell migration, apoptosis, cancer therapy

## Abstract

Human malignant melanoma is the most aggressive type of skin cancer with high metastatic ability. Despite several traditional therapies, the mortality rate remains high. *Lactobacillus plantarum* (*L. plantarum*), a species of lactic acid bacteria (LAB), is being studied for human health, including cancer treatment. However, few studies have elucidated the relationship between *L. plantarum* extract and human malignant melanoma. To investigate the effects of *L. plantarum* on human melanoma cells, A375 human melanoma cells were used and treated with *L. plantarum* L-14 extract. After the treatment, viability, migration ability, molecular changes of migration- and apoptosis-related genes, and the location of cytochrome c was evaluated. The L-14 extract inhibited the viability, migration of A375 cells as well as reduced expression of migration-related genes. In addition, it was confirmed that the L-14 extract induced intrinsic apoptosis in A375 cells. This study demonstrated that the L-14 extract exerted anticancer effects on A375 cells. Therefore, these data suggest that the L-14 extract is worth studying for the development of melanoma drugs using LAB.

## 1. Introduction

Cancer is the second leading cause of death globally, which humans have not yet fully conquered [1]. Skin cancer is a common form of cancer globally and its incidence rate has increased by 50 times in 70 years [2]. The World Health Organization attributes the cause of the elevated incidence rate to the increased exposure to ultraviolet (UV) to the decrease in ozone level or the use of UV-emitting tanning devices for cosmetic purposes. If a skin cancer is detected at an early stage and metastasis has not occurred, it usually be treated via surgery with a high survival rate [3]. However, when metastasis has progressed, the survival rate decrease dramatically, and chemotherapy and radiotherapy are applied with surgery [4]. Recently, targeted therapy and immunotherapy are used to improve the survival of patients with advanced melanoma that cannot be removed with surgery [5]. Approximately 80% of skin cancers are basal cell carcinoma, 16% are squamous cell carcinoma, and 4% are malignant melanoma [6]. While malignant melanoma has the smallest portion among skin cancers, it is responsible for most of the deaths related to skin cancer (more than 75%) because of its high metastatic and invasive abilities [7]. However, traditional therapies have harmful side effects such as the generation of reactive oxygen species or the toxicity to adjacent organs and tissues [8,9]. Therefore, the development of drugs, which can inhibit metastasis of melanoma and have fewer side effects, is required.

Lactic acid bacteria (LAB), which are derived from fermented foods are the most commonly used probiotics [10]. LAB are administered to humans for various purposes since they and their metabolites have beneficial effects [11]. They are generally considered safe if used at adequate doses [12]. Studies have shown that LAB and their metabolites modulate pathogen-induced immune response and enhanced innate immunity [13,14]. Pochard et al. also confirmed that LAB could mitigate allergic symptoms by reducing cytokine production [15]. *Lactobacillus plantarum* (*L. plantarum*) is a major species of LAB, and studies on the effects of *L. plantarum* on various cancers are being actively conducted [16,17]. However, little is known about the effects of *L. plantarum* on human malignant melanoma cells. Therefore, this study investigated whether *L. plantarum* in form of a cell- free extract has any effect on human malignant melanoma cells. After treating human malignant melanoma cells, A375P (low metastatic) and A375SM (high metastatic), with *L. plantarum* extract, the viability and molecular changes of the cells were analyzed.

## 2. Results

### 2.1. The L-14 Extract Affected the Growth and Viability of A375P and A375SM

**Figure 1:**
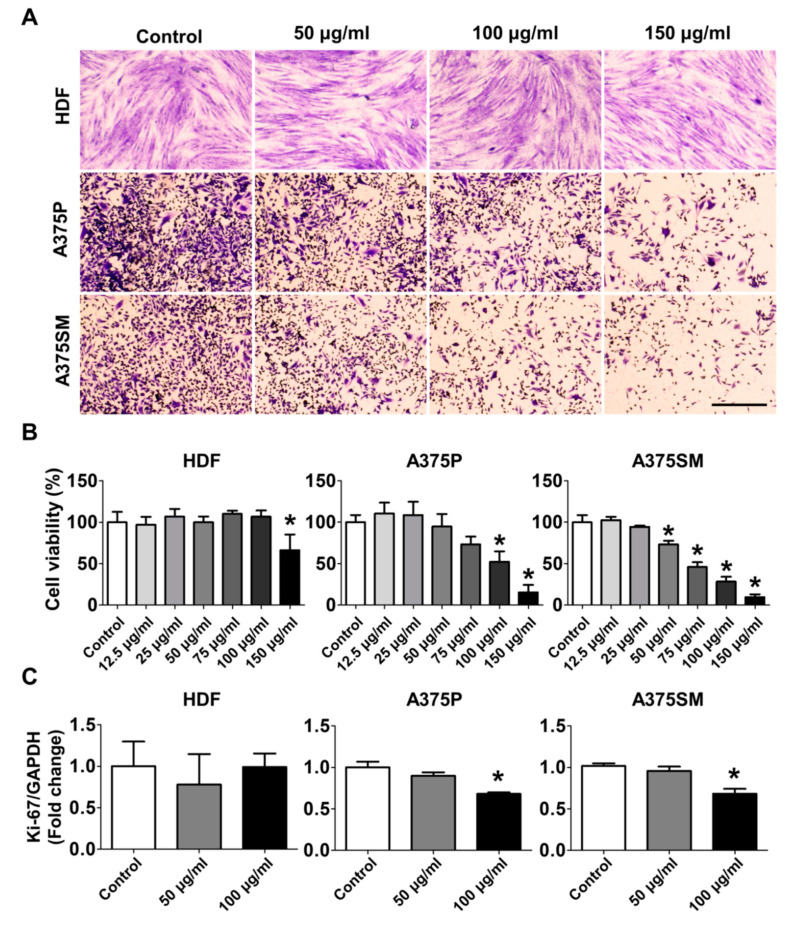
Effect of the L-14 extract on the growth of human dermal fibroblast (HDF) and human malignant melanoma cells, A375P and A375SM, after 72 h treatment of the L-14 extract. (**A**) The cells were stained with crystal violet solution. After staining with crystal violet solution, the damage by the L-14 extract was not affected by the L-14 extract in the three cells, whereas the growth of A375 cells was inhibited more than HDF. (**B**) Viability began to decrease in A375P and A375SM from lower concentrations in a dose-dependent manner than in HDF. (**C**) The mRNA expression of Ki-67 was reduced in 100 μg/mL of the L-14 extract treated group in both A375 cells but not in HDF. Scale bar = 200 μm. * *p* < 0.05 versus the control group.

**Figure 2:**
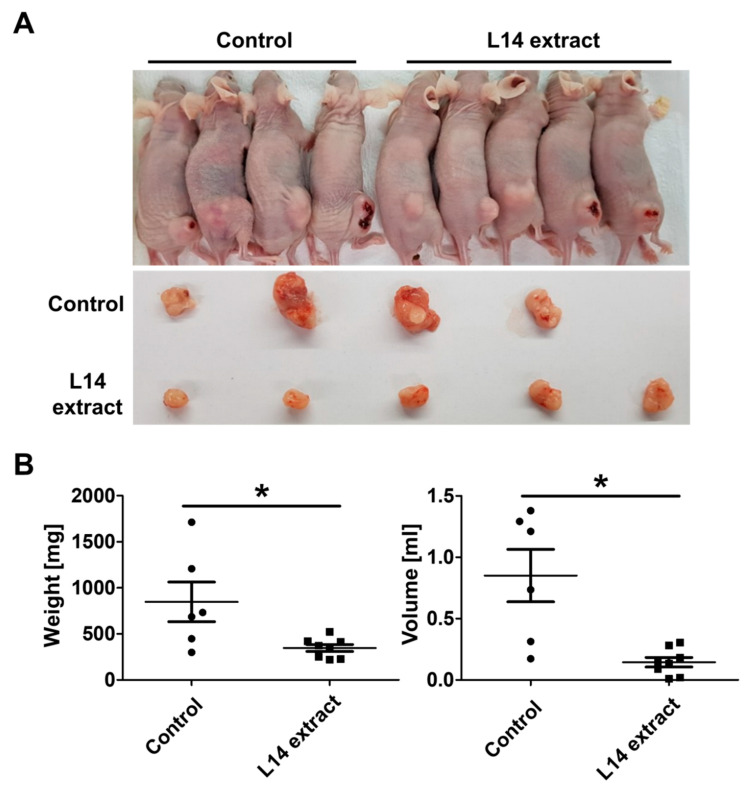
Inhibition of tumor growth by the L-14 extract in vivo. The tumor was induced by subcutaneously injecting A375SM cells (5 × 10^6^) into the right femoral region of immunodeficient BALB/c mice. After tumor growth, PBS (control group, n = 6) and the L-14 extract (500 mg/kg; n = 8) were treated intraperitoneally every two days for three weeks. (**A**) The tumors were smaller in the L-14 extract treated mice than in PBS treated mice. (**B**) The weight and volume of the tumors were quantified. * *p* < 0.05 versus the control group.

To investigate the effects of the L-14 extract on human skin-related cells, human dermal fibroblasts (HDF), A375P, and A375SM were treated with 50, 100, and 150 μg/mL L-14 extracts. After 72 h of treatment, the growth and appearance of the cells in the well were observed using the crystal violet solution (Figure 1A). The growth of HDF was decreased in the 150 μg/mL-treated group, whereas that of the A375 cells was dose-dependently inhibited in all treated groups. For quantitative analysis, the viability of the three types of cells was measured after 72 h of treatment by WST-8 viability assay (Figure 1B). Consistent with the staining assay, the viability of HDF was unchanged by the L-14 extract under 150 μg/mL while that of both A375 cells was significantly decreased in a dose-dependent manner. Based on the result of the viability assay, 50 μg/mL of the L-14 extract was determined to be the minimum concentration at which the growth of A375SM began to be inhibited and 100 μg/mL the maximum concentration at which the growth of HDF was not inhibited. The change of mRNA expression of Ki-67 confirmed the growth-inhibitory effect of the L-14 extract (Figure 1C). The treatment of 100 μg/mL L-14 extract significantly decreased the mRNA expression of Ki-67 in both A375 cells but not in HDF. The growth-inhibitory effect of the L-14 extract was also confirmed by the in vivo experiment. In the L-14 extract treated group, the tumors induced by subcutaneously injecting A375SM were evidently smaller than those of the control group (Figure 2A). The weight and volume of the tumors were quantified (Figure 2B).

### 2.2. The L-14 Extract Inhibited the Migration of A375P and A375SM

**Figure 3:**
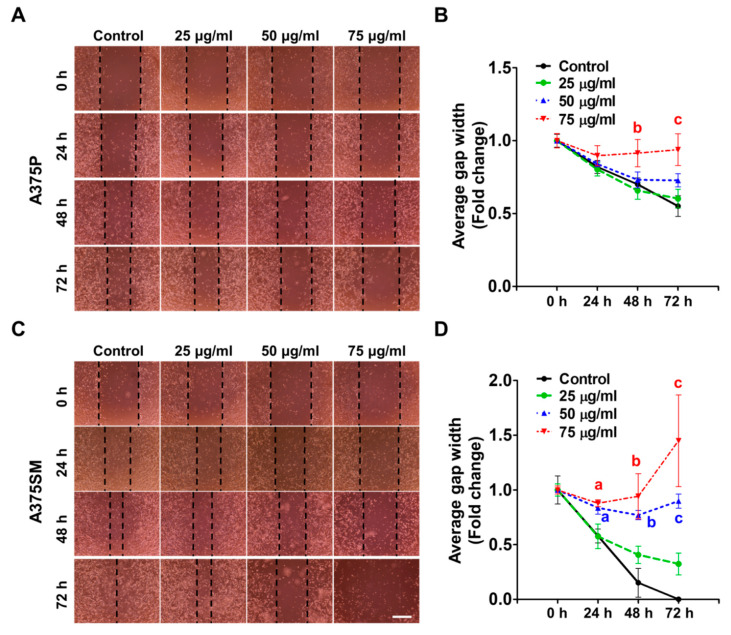
Analysis of cell migration ability after the treatment of the L-14 extract. The cells were cultured with the L-14 extract after scratching using a pipette tip. (**A**,**C**) The A375P cells were observed every 24 h after scratching and the treatment of the L-14 extract. (**B**,**D**) The average gap width was quantified. ^a,b^, and ^c^ *p* < 0.05 versus the control group at 24, 48, and 72 h, respectively. Scale bar = 200 μm.

To evaluate the anti-migratory effect of the L-14 extract on A375P and A375SM, the gap width was measured following the treatment of the L-14 extract. It was shown that migration was reduced in the L-14 extract-treated groups compared to the control group in a dose-dependent manner in both A375 (Figure 3A,C). The concentration of the L-14 extract was adjusted (25, 50, and 75 μg/mL) lower than other experiments since the anti-growth effect of the L-14 extract was promoted by mitomycin C (MMC), which is known as a DNA synthesis inhibitor. In the case of A375SM, the gap width of the 75 μg/mL-treated group was rather widened since the anti-migratory effect was added to the reduction of viability by the L-14 extract. The migration of cells was quantified and analyzed by measuring the average gap width (Figure 3B,D). The anti-migratory effect of the L-14 extract was more effective in A375SM than in A375P in a time- and dose-dependent manner. The gap was significantly less closed in 75 μg/mL L-14 extract treated group from 48 h compared to the control group in A375P. However, there was a significant difference in over 50 μg/mL L-14 extract treated groups from 24 h in A375SM.

### 2.3. The L-14 Extract Changed the mRNA and Protein Expression of Genes Associated with Metastasis of Cancers

**Figure 4:**
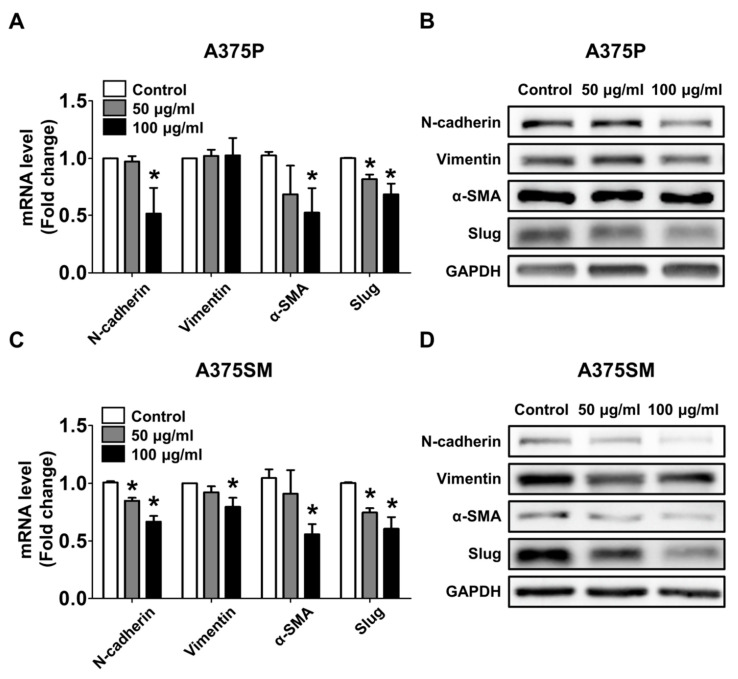
Changes in gene expression of migration-related genes after the treatment of the L-14 extract. (**A**,**B**) The mRNA and protein expression of N-cadherin, α-SMA, and Slug was decreased while mRNA of Vimentin had no significant change following the treatment of the L-14 extract in A375P. (**C**,**D**) The expression of all migration-related genes including Vimentin was downregulated in A37SM. Values of mRNA were normalized against GAPDH and depicted as fold change values. * *p* < 0.05 versus the control group.

The molecular changes in mesenchymal gene expression after the treatment were detected by quantitative real-time PCR (qRT-PCR) and Western blotting. The concentration of the L14-extract was determined as mentioned above (50 and 100 μg/mL). The mRNA and protein expression of mesenchymal cell markers including N-cadherin, alpha-smooth muscle actin (α-SMA), and Slug was decreased (Figure 4A,B). The expression of Vimentin only reduced in the protein level. In A375SM, both the mRNA and protein expression of mesenchymal cell markers were downregulated by the L-14 extract (Figure 4C,D).

### 2.4. Changes in mRNA and Protein Expression of Genes was Associated with Apoptosis

**Figure 5:**
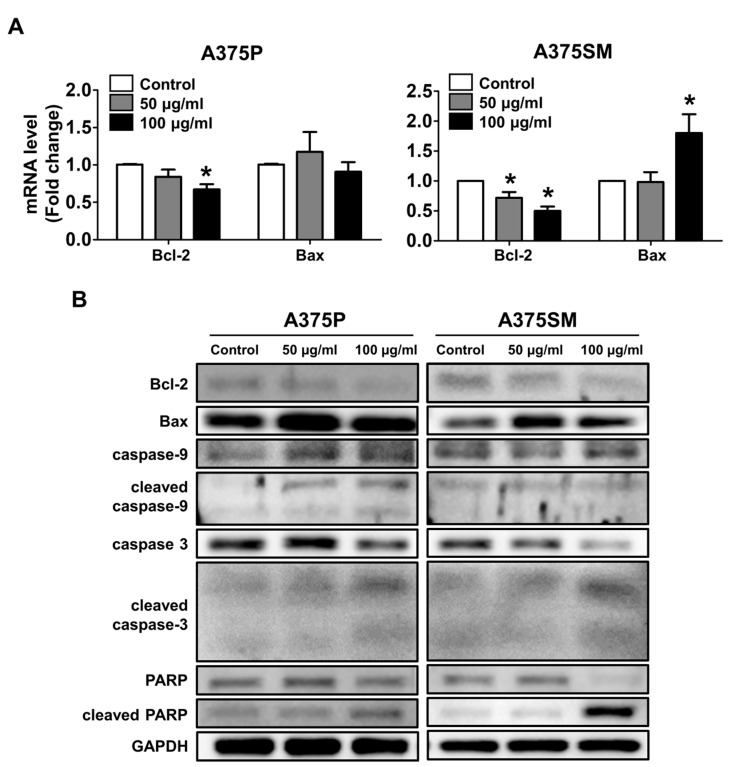
Expression of the apoptosis-related genes after the treatment of the L-14 extract. (**A**) The mRNA expression of Bcl-2 decreased significantly in both A375P and A375SM while the mRNA expression of Bax was reduced only in A375SM by the L-14 extract. (**B**) The protein expression of Bcl-2 and Bax was decreased and caspase-9, caspase-3, and PARP were cleaved. These results show that intrinsic apoptosis was induced in both A375 cells by the L-14 extract. Values of mRNA expression were normalized against GAPDH and depicted as fold change values. * *p* < 0.05 versus the control group.

To confirm whether the L-14 extract induced apoptosis in A375 cells, the apoptosis-related genes were analyzed after the treatment of the L-14 extract (50 and 100 μg/mL). The mRNA expression of Bcl-2, an anti-apoptotic marker, was significantly decreased by the L-14 extract in both A375 cells while that of Bax, an apoptosis regulator, was increased only in A375SM (Figure 5A). However, protein expression showed a reduced tendency in both A375 cells (Figure 5B). We observed a decrease in Bcl-2 protein, an increase in Bax protein, and activation of both caspase-9 and caspase-3. The L-14 extract also induced the cleavage of poly(ADP-ribose) polymerase (PARP), a protein involved in DNA repair.

### 2.5. Observation of the Release of Cytochrome C from the Mitochondria Following the Treatment with the L-14 Extract

**Figure 6:**
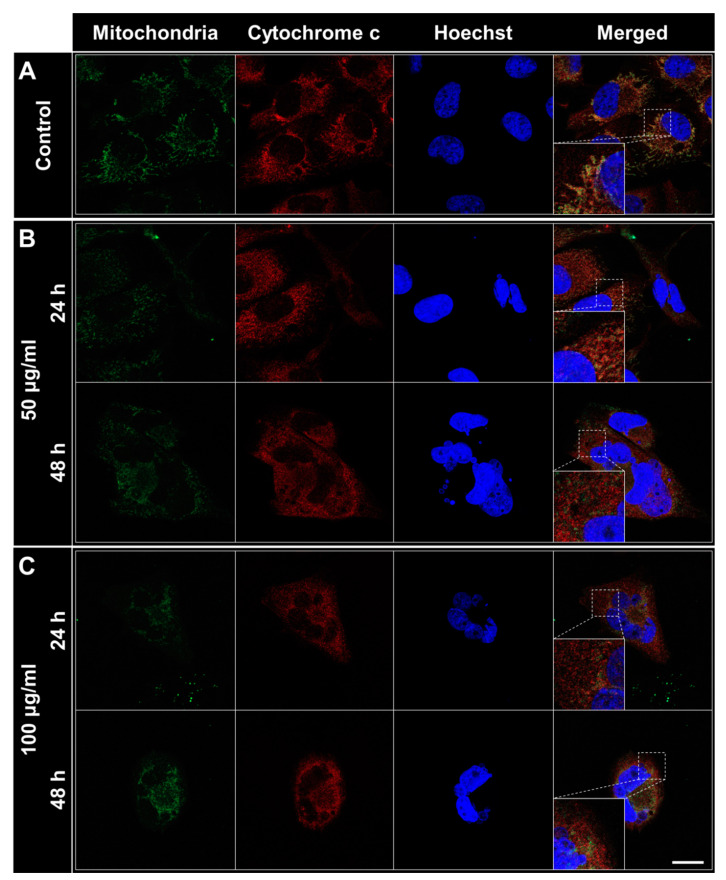
Immunofluorescence staining for the location of cytochrome c in A375SM after the treatment of the L-14 extract. The locational change of cytochrome c was detected by staining mitochondria (green) and cytochrome c (red). Hoechst indicates nucleus. In the L-14 extract treated group, the upper panels were at 24 h of the treatment and the lower panels were at 48 h of the treatment. (**A**) The non-treated cells did not show any sign of apoptosis. (**B**) The fragmentation of nucleus and the release of cytochrome c were induced from 48 h in the 50 μg/mL-treated group. (**C**) Apoptosis was induced from 24 h in the 100 μg/mL-treated group. Scale bar = 20 μm.

The process of the apoptotic pathway includes the release of cytochrome c from the mitochondrial intermembrane space to the cytosol. Therefore, the location of intracellular cytochrome c was probed by IF. The non-treated A375SM had an intact nuclear morphology and the same location of mitochondria and cytochrome c (Figure 6A). In the 50 μg/mL-treated group at 24 h of treatment, the morphological change of nucleus and the location of cytochrome c were not different from the control group while the fragmented nucleus and the release of cytochrome c were observed at 48 h (Figure 6B). Both characteristics were detected from 24 h in the group treated with 100 μg/mL (Figure 6C).

## 3. Discussion

Malignant melanoma is an aggressive cancer with a high metastatic ability. Despite the attempts to develop therapies for melanoma, the mortality rate remains high. For example, dacarbazine, the only approved melanoma chemotherapeutic drug by the Food and Drug Administration for patients with stage IV, does not increase the overall survival rate [18]. Although targeted therapy and immunotherapy are currently the most used types to treat melanoma, these therapies still have some limitations. Targeted therapy interferes with mutation of the BRAF gene, which stimulates cells to develop abnormally and divide out of control, but the mutation occurs in around half of all melanoma [19]. In the case of immunotherapy, inflammatory response can be caused in any of the organs of the body [20]. Therefore, the development of new drugs for melanoma is urgently required. LAB are widely used for the promotion of human health. Recently, there have been attempts to cure cancers using LAB [17]. However, the effects of *L. plantarum* extract on human malignant melanoma cells have not been investigated. Accordingly, this study was conducted to demonstrate the potential of *L. plantarum* as a treatment for human malignant melanoma using A375P and A375SM. The representative characteristics of cancers are unlimited proliferation, metastasis, and evasion of apoptosis [21]. The main objectives of various anticancer drugs are to hamper these characteristics of cancers and to return the cells to their normal state. We confirmed that the L-14 extract decreased the viability of human melanoma cells, A375P and A375SM, in vitro. In addition, the intraperitoneal injection of the L-14 extract significantly blocked the growth of A375SM-induced tumor in immunodeficient mice in vivo, indicating that the L-14 extract worked systemically. It could result from the downregulation of mRNA expression of Ki-67, a proliferation marker. It isn known that the expression of Ki-67 is strongly associated with the growth and proliferation of cancer, suggesting that it can be used as a marker of tumor malignancy. Studies have suggested that Ki-67 can be used as a tool for the diagnosis and prognosis of cancer [22,23].

In the next experiment, we confirmed that the L-14 extract had anti-migration effect on A375 cells. The inhibition of epithelial–mesenchymal transition (EMT), a process by which cells lose their epithelial junctions and gain the migratory and invasive ability, is important to the development of new drugs for cancer metastasis [24]. Studies have suggested that these mesenchymal genes are involved in the metastasis of cancer and can be therapeutic targets [25,26,27,28]. The treatment of the L-14 extract reduced the gene expression of mesenchymal markers, such as N-cadherin, Vimentin, α-SMA, and Slug [29]. Consistent with the gene expression, it was also confirmed that the L-14 extract inhibited the migration of both A375 cells at the cellular level.

The decrease in viability and expression of mesenchymal-related genes by the L-14 extract could be associated with the induction of apoptosis. It has been reported that the induction of EMT confers cancers with the resistance of apoptosis [30,31]. Apoptosis is typically characterized by nuclear fragmentation, changes of apoptosis-related gene expression, cleavage of apoptosis-related proteins, and locational change of cytochrome c. Apoptosis is divided into two major pathways, intrinsic and extrinsic pathways. In intrinsic or mitochondrial apoptosis, the released cytochrome c from mitochondria by Bax converts pro-caspase-9 into caspase-9 [32,33]. Subsequently, caspase-9 activates the executioner caspase-3, which thereafter induces the cleavage of PARP [34]. We confirmed the aforementioned features of intrinsic apoptosis after the treatment of the L-14 extract. In addition, it was detected that cytochrome c was released from the mitochondria to the cytosol, which has often been regarded as the point of no return in intrinsic apoptosis [35]. It is not confirmed whether the L-14 extract induces extrinsic apoptosis in A375 cells by these results. Therefore, further studies are required to find the pathway through which the L-14 extract induces apoptosis. However, melanoma drug resistance is due to the inhibition of the intrinsic apoptosis in general [36]. Thus, the induction of intrinsic apoptosis by the L-14 extract in A375 cells indicates that the L-14 extract could be used as a supplement with other drugs for melanoma. In the development of anticancer drugs, the induction of apoptosis is the most effective non-surgical method [37]. Although drugs to induce apoptosis have been actively studied, the traditional treatments have moderate-to-severe toxicities and side effects [38]. This study showed that the L-14 extract had less cytotoxicity on non-neoplastic cells, HDF, than both A375 cells, indicating that the L-14 extract could have fewer side effects on normal cells.

A375SM was more susceptible to the L-14 extract than A375P throughout this study. It is well-known that interleukin-8 (IL-8), which regulates proliferation and metastasis in melanoma, and its receptors, CXCR-1 and CXCR-2, are expressed more in A375SM than in A375P [39,40]. It was reported that downregulation of CXCR-1 and CXCR-2 inhibits proliferation, migration, and invasion in A375 [41]. In addition, integrin α_v_β_3_ is differentially expressed between A375P and A375SM [42]. Apoptosis can be stimulated by loss of integrin-mediated attachment to the extracellular matrix via the action of caspases [43]. The L-14 extract may affect the IL-8, CXCR-1, CXCR-2 or integrin of A375 cells, causing the difference in efficiency of the L-14 extract. Thus, in further study, it is necessary to confirm where the differences of efficiency occur.

## 4. Materials and Methods

### 4.1. Materials

Fetal bovine serum (FBS) and penicillin/streptomycin (P/S) were purchased from HyClone (Logan, UT, USA) and Gibco (Grand Island, NY, USA), respectively. Antibodies were purchased from the following companies: N-cadherin, Slug, Bax, caspase-3, and cleaved caspase-3 from Cell Signaling Technology (Danvers, MA, USA); α-SMA and Vimentin from Abcam (Cambridge, UK); caspase-9 and cleaved caspase-9 from Cusabio (Wuhan, China); PARP and cleaved PARP from Millipore (Burlington, MA, USA); Bcl-2 from Novus Biologicals (Centennial, CO, USA); and GAPDH from BioLegend (San Diego, CA, USA).

### 4.2. Cell Culture

HDF were provided by the American Type Culture Collection (Manassas, VA, USA). A375P and A375SM were purchased from the Korean Cell Line Bank (Seoul, Korea). HDF and both A375 cells were cultured in Dulbecco’s Modified Eagle Medium (WELGENE, Daegu, Korea) with 10% FBS and 1% P/S at 37 °C with 5% CO_2_, and the culture medium was replaced every 2 days.

### 4.3. Preparation of L. Plantarum Extract

The *L. plantarum* L-14 strain (KTCT13497BP) used in this study was provided from NeoRegen Biotech (Suwon, Gyeonggi-do, Korea) and cultured in sterilized de Man, Rogosa and Sharpe (MRS; Hardy Diagnostics, Santa Maria, CA, USA) broth at 37 °C for 18 h. The cultured L-14 was centrifuged at 10,000× *g* for 15 min at 4 °C for harvest. The L-14 pellet was washed with distilled water (DW) three times to remove the MRS broth medium. After washing, the L-14 pellet was resuspended with DW and sonicated for 30 min on ice. The sonicated L-14 was centrifuged at 12,000× *g* for 15 min at 4 °C. The supernatant was filtered using a 0.45 μm filter (Sartorius, Göttingen, Germany) and lyophilized for 72 h. The extract was dissolved at 50 mg/mL in phosphate-buffered saline (PBS) and filtered using a 0.45 μm filter for the following experiments.

### 4.4. Cell Viability Assay

HDF, A375P and A375SM were seeded at a density of 2.0 × 10^3^ cells per well in 96-well plates and allowed to adhere for 24 h. The medium was replaced with various concentrations of the L-14 extract and maintained. After 72 h, the effects of the extract on cell growth were determined by Quanti-Max WST-8 cell viability kit (BIOMAX, Seoul, Korea).

### 4.5. qRT-PCR

Total RNA was extracted from A375P and A375SM following 24 h treatment of the L-14 extract at a concentration of 50 and 100 μg/mL using MiniBEST Universal RNA Extraction Kit (TaKaRa Bio Inc., Shiga, Japan). The isolated RNA was reverse transcribed to cDNA using M-MLV Reverse Transcriptase (Promega, Madison, WI, USA). Each gene was amplified and analyzed with TB Green Premix Ex Taq II (TaKaRa) and StepOnePlus (Applied Biosystems, Foster City, CA, USA). Primer sequences for qRT-PCR are listed in Table 1. The reagents were used according to the manufacturer’s recommendations. GAPDH was used as an endogenous control and for normalization of the differences in individual samples.

### 4.6. Animals and In Vivo Anti-Tumor Experiments

All animal experiments were performed according to the guidelines of the Institutional Animal Care and Use Committee of Seoul National University (approval number: SNU-161024-3). To induce tumors in mice, 5 × 10^6^ cells of A375SM were subcutaneously injected into the right femoral region of immunodeficient BALB/c mice (n = 14). After 5 days for tumor growth, tumor-induced mice were intraperitoneally treated with PBS (n = 6) and the L-14 extract (500 mg/kg, n = 8) every 2 days. Following treatment for 3 weeks, the mice were sacrificed. The tumors were then dissected and analyzed. Its volume was calculated using the formula (length × width^2^)/2.

### 4.7. Cell Migration Assay

Both A375 cells were seeded in 12-well plates and cultured until they reached approximately 80% confluence. Then, both A375 cells were treated with MMC (Cayman, Ann Arbor, MI, USA) for 3 h before the cell migration assay to exclude the effect of proliferation. A scratch was drawn in the center of the well using a pipette tip. Each well was washed by PBS three times to remove MMC and detached cells. A375 cells were treated with various concentrations of the L-14 extract. The scratched monolayer of cells was photographed via EVOS XL Core microscope (Life Technologies, Carlsbad, CA, USA) at 24, 48, and 72 h of treatment at 40× magnification.

### 4.8. Protein Isolation and Western Blot

Both A375 cells were seeded at a density of 5 × 10^4^ cells per well in 6-well plates and stabilized for 24 h. Then, the cells were treated with various concentrations of the L-14 extract for 72 h. Total proteins were extracted by Passive lysis buffer (Promega) containing protease inhibitor and centrifuged at 12,000× *g* for 15 min at 4 °C to remove any insoluble debris. An equal amount (10–20 μg) of proteins in the supernatants was loaded, isolated by performing sodium dodecyl sulfate–polyacrylamide gel electrophoresis, and transferred to polyvinylidene difluoride membranes. After blocking for 1 h at room temperature (RT), the proteins were probed with primary antibodies overnight at 4 °C followed by washing with 0.1% Tween 20 (Sigma-Aldrich, Saint Louis, MO, USA) in tris-buffered saline. The primary antibodies were detected horseradish-peroxidase-conjugated secondary antibodies for 1 h at RT, which were visualized using ECL Western Blotting Substrate (Daeil Lab Service, Seoul, Korea).

### 4.9. Immunofluorescence (IF) Staining for the Detection of Cytochrome C Release

A375SM was seeded at a density of 3 × 10^3^ cells in a circular coverslip pre-coated with gelatin in 24-well plates. After 24 h of stabilization, the cells were treated with various concentrations of the L-14 extract. Following 24 and 48 h of treatment, the culture medium was replaced with fresh medium containing 100 nM MitoTracker (Invitrogen, Carlsbad, CA, USA). The cells were incubated for 30 min at 37 °C and washed with PBS. IF staining was performed according to standard protocols. Briefly, washed cells on the coverslip were fixed with 4% paraformaldehyde, permeabilized with 0.15% Triton X-100 (Sigma), blocked with 3% bovine serum albumin (Bovogen, East Keilor, Australia), probed by anti-cytochrome c antibody (Novus Biologicals) and visualized using Alexa Fluor 647-conjugated secondary (Abcam). The coverslip was mounted with Hoechst 33,342 (Invitrogen). All samples were scanned via LSM 800 confocal laser scanning microscope (Carl Zeiss, Oberkochen, Germany) at 630× magnification.

### 4.10. Statistical Analysis

All values in this study were obtained from three independent experiments in triplicate and displayed as the mean ± standard deviation. Results of *p* < 0.05 was considered statistically significant.

## 5. Conclusions

We demonstrated that the L-14 extract decreased migratory ability via reduction of mesenchymal cell-related gene expression and induced intrinsic apoptosis by upregulating Bax; downregulating Bcl-2; cleaving caspase-9, caspase-3, and PARP; and releasing cytochrome c from the mitochondria in human malignant cells A375. While showing these anticancer effects, the L-14 extract had less cytotoxicity to non-neoplastic cells, HDF. Further studies are required to find the effect molecule via fractionation for the determination of doses and clinical trials and to investigate how the L-14 extract induces apoptosis. Although it was not confirmed that the L-14 extract has anti-cancer effects on different melanoma cell lines or primary melanoma cells, the results in this study indicate that the L-14 extract is worthwhile to research for the treatment of melanoma.

## Figures and Tables

**Table 1:** Primer sequence used for qRT-PCR.

Gene		Sequence (5′-3′)	Product Size (bp)
Ki-67	Forward	AGT TTG CGT GGC CTG TAC TAA	202
	Reverse	AGA AGA AGT GGT GCT TCG GAA	
N-cadherin	Forward	ACA GTG GCC ACC TAC AAA GG	201
	Reverse	CCG AGA TGG GGT TGA TAA TG	
Vimentin	Forward	ATC CAA GTT TGC TGA CCT CTC TGA	99
	Reverse	GAC TGC ACC TGT CTC CGG TAC TC	
α-SMA	Forward	GAC GTA CAA CTG GTA TTG TG	144
	Reverse	TCA GGA TCT TCA TGA GGT AG	
Slug	Forward	TTG TGG CCT TCT TTG AGT TCG GTG	146
	Reverse	GGT GCC TCA GGT ACT CAG TCA	
Bcl-2	Forward	TTG TGG CCT TCT TTG AGT TCG GTG	111
	Reverse	GGT GCC TCA GGT ACT CAG TCA	
Bax	Forward	CCT GTG CAC CAA GGT GCC GGA ACT	99
	Reverse	CCA CCC TGG TCT TGG ATC CAG CCC	
GAPDH	Forward	CGC TGA GTA CGT CGT GGA GTC	172
	Reverse	GCT GAT GAT CTT GAG GCT GTT GTC	

## References

[1] Van der Walt N.B., Zakeri Z., Cronje M.J. (2016). The induction of apoptosis in A375 malignant melanoma cells by *Sutherlandia frutescens*. Evid. Based Complement. Alternat. Med..

[2] Guy G.P., Ekwueme D.U. (2011). Years of potential life lost and indirect costs of melanoma and non-melanoma skin cancer: A systematic review of the literature. Pharmacoeconomics.

[3] Balch C.M., Gershenwald J.E., Soong S.J., Thompson J.F., Atkins M.B., Byrd D.R., Buzaid A.C., Cochran A.J., Coit D.G., Ding S. (2009). Final version of 2009 AJCC melanoma staging and classification. J. Clin. Oncol..

[4] Song X., Zhao Z., Barber B., Farr A.M., Ivanov B., Novich M. (2015). Overall survival in patients with metastatic melanoma. Curr. Med. Res. Opin..

[5] Aris M., Barrio M.M. (2015). Combining immunotherapy with oncogene-targeted therapy: A new road for melanoma treatment. Front. Immunol..

[6] Freeman K., Dinnes J., Chuchu N., Takwoingi Y., Bayliss S.E., Matin R.N., Jain A., Walter F.M., Williams H.C., Deeks J.J. (2020). Algorithm based smartphone apps to assess risk of skin cancer in adults: Systematic review of diagnostic accuracy studies. Br. Med. J..

[7] Faiao-Flores F., Coelho P.R., Arruda-Neto J., Maria D.A. (2011). Boron neutron capture therapy induces cell cycle arrest and DNA fragmentation in murine melanoma cells. Appl. Radiat. Isot..

[8] Ozben T. (2015). Antioxidant supplementation on cancer risk and during cancer therapy: An update. Curr. Top. Med. Chem..

[9] Cosentino D., Piro F. (2018). Hyaluronic acid for treatment of the radiation therapy side effects: A systematic review. Eur. Rev. Med. Pharmacol. Sci..

[10] Zielinska D., Kolozyn-Krajewska D. (2018). Food-origin lactic acid bacteria may exhibit probiotic properties: Review. Biomed. Res. Int..

[11] Ren D., Zhu J., Gong S., Liu H., Yu H. (2018). Antimicrobial characteristics of lactic acid bacteria isolated from homemade fermented foods. Biomed. Res. Int..

[12] Bintsis T. (2018). Lactic acid bacteria: Their applications in foods. J. Bacteriol. Mycol..

[13] Perdigon G., Alvarez S., Rachid M., Aguero G., Gobbato N. (1995). Immune system stimulation by probiotics. J. Dairy. Sci..

[14] Corr S.C., Gahan C.G., Hill C. (2007). Impact of selected *Lactobacillus* and *Bifidobacterium* species on Listeria monocytogenes infection and the mucosal immune response. FEMS Immunol. Med. Microbiol..

[15] Pochard P., Gosset P., Grangette C., Andre C., Tonnel A.B., Pestel J., Mercenier A. (2002). Lactic acid bacteria inhibit TH2 cytokine production by mononuclear cells from allergic patients. J. Allergy Clin. Immunol..

[16] Kassayova M., Bobrov N., Strojny L., Orendas P., Demeckova V., Jendzelovsky R., Kubatka P., Kiskova T., Kruzliak P., Adamkov M. (2016). Anticancer and immunomodulatory effects of *Lactobacillus plantarum* LS/07, inulin and melatonin in NMU-induced rat model of breast cancer. Anticancer. Res..

[17] Chuah L.O., Foo H.L., Loh T.C., Mohammed Alitheen N.B., Yeap S.K., Abdul Mutalib N.E., Abdul Rahim R., Yusoff K. (2019). Postbiotic metabolites produced by *Lactobacillus plantarum* strains exert selective cytotoxicity effects on cancer cells. BMC Complement. Altern. Med..

[18] Yue Y., Liu L., Liu P., Li Y., Lu H., Li Y., Zhang G., Duan X. (2020). Cardamonin as a potential treatment for melanoma induces human melanoma cell apoptosis. Oncol. Lett..

[19] Long G.V., Menzies A.M., Nagrial A.M., Haydu L.E., Hamilton A.L., Mann G.J., Hughes T.M., Thompson J.F., Scolyer R.A., Kefford R.F. (2011). Prognostic and clinicopathologic associations of oncogenic BRAF in metastatic melanoma. J. Clin. Oncol..

[20] Escandon Brehm J., Bedogni B. (2020). Blockade of CCR5 in melanoma: An alternative immune checkpoint modulator. Exp. Dermatol..

[21] Fouad Y.A., Aanei C. (2017). Revisiting the hallmarks of cancer. Am. J. Cancer Res..

[22] Li L.T., Jiang G., Chen Q., Zheng J.N. (2015). Ki67 is a promising molecular target in the diagnosis of cancer (review). Mol. Med. Rep..

[23] Brown D.C., Gatter K.C. (2002). Ki67 protein: The immaculate deception?. Histopathology.

[24] Boyer B., Thiery J.P. (1993). Epithelium-mesenchyme interconversion as example of epithelial plasticity. APMIS..

[25] Wels C., Joshi S., Koefinger P., Bergler H., Schaider H. (2011). Transcriptional activation of ZEB1 by Slug leads to cooperative regulation of the epithelial-mesenchymal transition-like phenotype in melanoma. J. Invest. Dermatol..

[26] Lee H.W., Park Y.M., Lee S.J., Cho H.J., Kim D.H., Lee J.I., Kang M.S., Seol H.J., Shim Y.M., Nam D.H. (2013). Alpha-smooth muscle actin (ACTA2) is required for metastatic potential of human lung adenocarcinoma. Clin. Cancer Res..

[27] Mrozik K.M., Blaschuk O.W., Cheong C.M., Zannettino A.C.W., Vandyke K. (2018). N-cadherin in cancer metastasis, its emerging role in haematological malignancies and potential as a therapeutic target in cancer. BMC Cancer.

[28] Li M., Zhang B., Sun B., Wang X., Ban X., Sun T., Liu Z., Zhao X. (2010). A novel function for vimentin: The potential biomarker for predicting melanoma hematogenous metastasis. J. Exp. Clin. Cancer Res..

[29] Zeisberg M., Neilson E.G. (2009). Biomarkers for epithelial-mesenchymal transitions. J. Clin. Investig..

[30] Gotzmann J., Mikula M., Eger A., Schulte-Hermann R., Foisner R., Beug H., Mikulits W. (2004). Molecular aspects of epithelial cell plasticity: Implications for local tumor invasion and metastasis. Mutat. Res..

[31] Chang J.C. (2016). Cancer stem cells: Role in tumor growth, recurrence, metastasis, and treatment resistance. Medicine.

[32] Jiang X., Wang X. (2000). Cytochrome c promotes caspase-9 activation by inducing nucleotide binding to Apaf-1. J. Biol. Chem..

[33] Liu X., Kim C.N., Yang J., Jemmerson R., Wang X. (1996). Induction of apoptotic program in cell-free extracts: Requirement for dATP and cytochrome c. Cell.

[34] Tewari M., Quan L.T., O’Rourke K., Desnoyers S., Zeng Z., Beidler D.R., Poirier G.G., Salvesen G.S., Dixit V.M. (1995). Yama/CPP32 beta, a mammalian homolog of CED-3, is a CrmA-inhibitable protease that cleaves the death substrate poly(ADP-ribose) polymerase. Cell.

[35] Ferraro E., Pulicati A., Cencioni M.T., Cozzolino M., Navoni F., di Martino S., Nardacci R., Carri M.T., Cecconi F. (2008). Apoptosome-deficient cells lose cytochrome c through proteasomal degradation but survive by autophagy-dependent glycolysis. Mol. Biol. Cell.

[36] Mohana-Kumaran N., Hill D.S., Allen J.D., Haass N.K. (2014). Targeting the intrinsic apoptosis pathway as a strategy for melanoma therapy. Pigment. Cell Melanoma Res..

[37] Pfeffer C.M., Singh A.T.K. (2018). Apoptosis: A target for anticancer therapy. Int. J. Mol. Sci..

[38] Wu S., Singh R.K. (2011). Resistance to chemotherapy and molecularly targeted therapies: Rationale for combination therapy in malignant melanoma. Curr. Mol. Med..

[39] Gutman M., Singh R.K., Xie K., Bucana C.D., Fidler I.J. (1995). Regulation of interleukin-8 expression in human melanoma cells by the organ environment. Cancer Res..

[40] Varney M.L., Li A., Dave B.J., Bucana C.D., Johansson S.L., Singh R.K. (2003). Expression of CXCR1 and CXCR2 receptors in malignant melanoma with different metastatic potential and their role in interleukin-8 (CXCL-8)-mediated modulation of metastatic phenotype. Clin. Exp. Metastasis.

[41] Shang F.M., Li J. (2019). A small-molecule antagonist of CXCR1 and CXCR2 inhibits cell proliferation, migration and invasion in melanoma via PI3K/AKT pathway. Med. Clin..

[42] Gehlsen K.R., Davis G.E., Sriramarao P. (1992). Integrin expression in human melanoma cells with differing invasive and metastatic properties. Clin. Exp. Metastasis.

[43] Stupack D.G., Cheresh D.A. (2004). A Bit-role for integrins in apoptosis. Nat. Cell Biol..

